# Dietary Supplementations as Neuroprotective Therapies: Focus on NT-020 Diet Benefits in a Rat Model of Stroke

**DOI:** 10.3390/ijms13067424

**Published:** 2012-06-15

**Authors:** Yuji Kaneko, Lourdes Cortes, Cyndy Sanberg, Sandra Acosta, Paula C. Bickford, Cesar V. Borlongan

**Affiliations:** 1 Department of Neurosurgery and Brain Repair, University of South Florida College of Medicine, 12901 Bruce B Downs Blvd, Tampa, FL 33612, USA; E-Mails: ykaneko@health.usf.edu (Y.K.); lcortes1@health.usf.edu (L.C.); sacosta@health.usf.edu (S.A.); pbickfor@health.usf.edu (P.C.B.); 2 Natura Therapeutics, Inc., Tampa, FL 33612, USA; E-Mail: cds@naturatherapeutics.com; 3 James A Haley Veterans Hospital, Research Service, Tampa, FL 33612, USA

**Keywords:** herbal extracts, cerebral ischemia, prophylactic, therapeutic window, mechanism

## Abstract

Stroke remains the number one cause of disability in the adult population. Despite scientific progress in our understanding of stroke pathology, only one treatment (tissue plasminogen activator or tPA) is able to afford benefits but to less than 3% of ischemic stroke patients. The development of experimental dietary supplement therapeutics designed to stimulate endogenous mechanisms that confer neuroprotection is likely to open new avenues for exploring stroke therapies. The present review article evaluates the recent literature supporting the benefits of dietary supplementation for the therapy of ischemic stroke. This article focuses on discussing the medical benefits of NT-020 as an adjunct agent for stroke therapy. Based on our preliminary data, a pre-stroke treatment with dietary supplementation promotes neuroprotection by decreasing inflammation and enhancing neurogenesis. However, we recognize that a pre-stroke treatment holds weak clinical relevance. Thus, the main goal of this article is to provide information about recent data that support the assumption of natural compounds as neuroprotective and to evaluate the therapeutic effects of a dietary supplement called NT-020 as in a stroke model. We focus on a systematic assessment of practical treatment parameters so that NT-020 and other dietary supplementations can be developed as an adjunct agent for the prevention or treatment of chronic diseases. We offer rationale for determining the optimal dosage, therapeutic window, and mechanism of action of NT-020 as a dietary supplement to produce neuroprotection when administered immediately after stroke onset. We highlight our long-standing principle in championing both translational and basic science approaches in an effort to fully reveal the therapeutic potential of NT-020 as dietary supplementation in the treatment of stroke. We envision dietary supplementation as an adjunct therapy for stroke at acute, subacute, and even chronic periods.

## 1. Introduction

Stroke is the number one cause of disability in the adult population [1]. Despite intense laboratory investigations and clinical trials, currently no satisfactory treatment is available to prevent the neuronal death and neurological dysfunction in stroke patients. An understanding of the endogenous mechanisms that confer neuroprotection is key in order to develop stroke therapies [2]. This review paper aims to explore and evaluate the therapeutic and medical benefits of dietary supplementations. The review focuses on the benefits of a dietary supplementation called NT-020 for the prevention and treatment of a ischemic stroke model in rats. The present review article also evaluates the recent literature supporting the benefits of dietary supplementation for the therapy of ischemic stroke [3,4]. This article focuses on discussing the medical benefits of NT-020 as an adjunct agent for stroke therapy.

Currently, research on dietary supplementation to alleviate vascular dieases, ischemic damage and to increase neuronal plasticity is gaining popularity among laboratories that seek a reliable therapy to prevent neurodegeneration or to even slow the onset of brain pathologies. There is a well supported body of literature about the positive medicinal effects of dietary supplementation. In a review article by Arab and Liebeskird, epidemiologic studies were evaluated to clarify the effects of dietary supplementation of tea catechins in animal models of stroke and in humans. The results showed that across studies, there were highly consistent neuroprotective effects when ingesting tea catechins prior or shortly after ischemic insult. Ingestion of tea catechins was effective in reducing stroke volume after the ischemic insults in all subjects including humans [5]. Moreover, in a lipopolysaccharide (LPS) induced inflammation study, the effects of natural compounds on chronic inflammation in the brain millieu were evaluated. In these experiments, rats were supplemented with 0.1% of a blue-green algae also known as spirulina for about 30 days in order to evaluate their anti-inflammatory and neuroprotective effects. Evaluation of hippocampal granular cells showed that there was a profound decrease of dividing cells in the subgranular cell layer of the dendate gyrus after LPS insult in the control diet group, relative to the spirulina enriched diet. The supplementation of spirulina seemed to negate the inflammatory damaging effect of LPS insult [6]. It is imperative to recognize that more research needs to be done to determine the mechanism of action of dietary supplementation, but it is also imperative to recognize that supplementations of nutrients previous to, during, or after disease state holds a promising tactic when talking about diseases with multiple etiologies and poor prognosis such as cardiovascular, stroke, and neurodegenerative diseases.

### 1.1. Patient Numbers and Current Methods of Care for Stroke

The treatment of stroke represents current unmet medical conditions of significance around the world. The therapy for stroke is limited. Other than one recombinant protein therapy directed at the dissolution of thrombi in affected blood vessels in adults following stroke, tumor plasminogen activator or tPA, no specific treatment is available for either focal cerebral ischemia or global ischemic event. Small molecule therapies such as anti-platelet drugs, anti-coagulants, and statins act as prophylactics and have no immediate benefit following an acute attack [2]. The numbers of affected individuals, the costs necessary to facilitate their care and rehabilitation, coupled with the lack of therapies, warrant significant research efforts to alleviate the suffering of stroke victims. Stroke is the third leading cause of death and the leading cause for disability in the United States. In 2004, stroke had a combined direct and indirect cost of over 53 billion dollars in the United States alone. The mean lifetime cost of ischemic stroke to a single patient in the United States is estimated at 140,048 dollars; this includes inpatient care, rehabilitation, and follow-up care necessary for lasting deficits. Approximately two out of every 1000 adults will have their first stroke in any given year in the United States [7].

### 1.2. Dietary Supplementation and Neurogenesis

Until recently, the non-regenerative capability of the adult damaged brain was an accepted scientific dogma. However, accumulating evidence over the last decades indicates that neurogenesis occurs during adulthood, and that neurons and astrocytes can be generated from isolated cells of the adult mammalian central nervous system (CNS) [8]. There are two areas where stem cells reside, namely the subventricular zone (SVZ) and the subgranular layer (SG) of the dentate gyrus (DG) of the hippocampus [9,10]. Studies on the functional organization of these neural stem cells (NSC) have demonstrated that NSC from the SVZ travel to the olfactory bulb through the rostral migratory stream (RMS) in where they are going to differentiate further into interneurons and astrocytes. NSCs present on the SG of the dentate gyrus will differentiate into granular cells and incorporate to the granular layer of the dentate gyrus of the hippocampus. Interestingly, this adult proliferation of NSC into the DG has been linked to neuronal plasticity associated with learning and memory [11,12]. Also, largely based on this phenomenon of neurogenesis and plasticity, several laboratory studies have examined stem cell therapy for treating various diseases in the CNS, including stroke, traumatic brain injury, and neurodegenerative diseases, such as Parkinson’s disease and Alzheimer’s disease. Stem cell therapy, however, remains as an experimental treatment [13–15]. While brain injury has been shown to trigger transient and limited neurogenesis, this endogenous protective mechanism is not capable of reversing the cell death cascade in the CNS [13–15]. It is, however, recognized that strategies designed to enhance the endogenous neurogenesis and neuronal plasticity are potentially beneficial for treating brain disorders [13–15].

Regenerative medicine has emerged as a new scientific field advancing stem cell therapy for treating brain disorders, with emphasis on either transplanting exogenous stem cells or amplifying endogenous stem cells via dietary suppplementation [16,17]. Our long-standing research interest is in the latter one, which incorporates a natural way of protecting, and possibly repairing the damaged brain and correcting the neurological impairments [3,6,7,18–20]. This review article will elucidate the biological effects of supplementation of “nutritious diet extracts/compounds”, referred to herein as NT-020, also called NutraStem^®^, which is a proprietary blend plus individual chemicals such as vitamin D3 (Natura Therapeutics, Tampa, FL, USA). NT-020 is an anti-inflammatory, antioxidant proprietary formula that contains polyphenols from blueberry extract, catechins from green tea, aminoacids such as carnosine, and vitamin D3. As a nutritious diet formula, it was shown previously to act synergistically to promote neurogenesis in adult rats and to exert functional benefits in a stroke model [3,21]. By “nutritious” diets, we refer to diets that are supplemented with nutrients such as antioxidant compounds and essential fatty acids that organisms can use to regulate their metabolic processes and balance homeostasis [22]. Studies have shown that fruits and vegetables contain significant amounts of antioxidants such as carotenoids, flavonoids, and tocopherols that may reduce the risk of developing neurodegenerative diseases [3,23]. In 2009, Valente and colleagues demonstrated that a diet rich in polyphenols and polyunsaturated fatty acids could positively influence neurogenesis in the adult mice. In their studies, Valente and colleagues refer to this diet as the LMN diet. After feeding mice with the LMN diet, an increase in cell proliferation, immature neurons, and differentiated neurons expressing tyrosine hydroxylase, calretinin and calbindin was found in brain of these mice. Specifically, an increase of proliferation and immature neurons was found on the subgranular cell layer of the dentate gyrus of the hippocampus. Likewise, an increase in numbers of proliferating cells expressing immature and mature neuronal markers was found on the SVZ of the lateral ventricle and migration towards the olfactory bulb. Interestingly, there was also noted an increase of astrocytes and oligodendrocytes through the rostral migratory stream and the olfactory bold. The neuroprotective effect of the LMN diet was tested *in vitro*. Primary cell neurons were cultured with LMN cream, hydrogen peroxide, and Aβ1–42 to asses the antioxidant benefits against toxic insults. Results by western blot analysis revealed that the LMN diet salvages cell death associated with either hydrogen peroxide or toxic amyloid beta insult [24]. In a different study, neuroprotection was demonstrated in mice after chronic supplemention of omega-3 polyunsaturated fatty acids (PUFA). It was shown that after six weeks of chronic supplementation with PUFA alone, combined with anti-depressant drugs such as alpha-linolenic acid or imipramine, mice showed a positive increase on both swimming and climbing behaviors. Also, it was noticed that supplementation of PUFA and anti-depressant drugs may be responsible for modulating brain plasticity in these mice. A significant increase of proliferating cells was found in the hippocampus along with an increase of hippocampal volume. Protein expression analysis showed that synaptophisin and BDNF were also elevated relative to control mice. This study supports the notion of essential fatty acids as a dietary supplement with medicinal potential as neuroprotective and antidepressant adjunct therapy [25].

Interestingly, the reverse model of diet restriction and even fasting has been recently reported to influence neurogenesis [26,27]. In particular, caloric manipulation has been shown to increase neurogenesis in CNS neurogenic sites, which coincided with amelioration of stroke-induced behavioral and histological deficits [28–30]. Careful examination of the diet restriction regimen reveals that the overarching principle of such neurogenesis-enhancing strategy is to eliminate the “unhealthy” or unwanted calories. We propose here that an equally robust neuroprotective approach may be achieved by the reverse paradigm of unhealthy diet restriction, in that increasing the “nutritious” diet should also render a therapeutically potent neurogenesis. Hence, we put forward the unifying concept of diet manipulation that encompasses either eliminating the unhealthy diet or enhancing the nutritious diet in order to achieve a reliable therapy for neuroprotection before and after ischemic stroke.

### 1.3. Recent Studies on Diet Supplementation Therapy in Ischemic Injury Models

Wang and colleagues [31] recently demonstrated the neuroprotective effects of diets supplemented in blueberry, spinach, and spirulina in focal ischemic brain. In this study, they fed adult male Sprague-Dawley rats with equal amounts of the diets (blueberry, spinach, and spirulina) or with control diet over a 4-week period, and thereafter subjected the animals to stroke surgery involving transient ligation of the middle cerebral artery (MCAl). This MCAl stroke model produces consistent cerebral infarction predominantly to the cortex. Behavioral and histological assays at eight and 48 h revealed that stroke animals which received blueberry, spinach, or spirulina enriched diets displayed significant increase in locomotor activity that coincided with reduction in the volume of infarction in the cerebral cortex. Analyses of physiological parameters revealed no differences in blood biochemistry, blood CO_2_, and electrolyte levels across groups, suggesting that changes in physiological functions did not contribute to the observed neuroprotection. In addition, stroke animals treated with blueberry, spinach, or spirulina exhibited significantly lower caspase-3 activity in the ischemic hemisphere. These data demonstrate that diet supplementation with blueberry, spinach, or spirulina attenuated stroke-induced cerebral infarction and apoptosis, and general locomotor impairments. However, the exact mechanism underlying such neuroprotection remains to be determined. Although not in a stroke model, short-term supplementation with blueberry (one of the components of NT-020) has been shown to increase hippocampal neurogenesis [32], which could account for the improvement of cognitive function of aged animals treated with this supplement [23].

Recent studies on dietary supplementation have focused their attention on essential fatty acids for the prevention and treatment of cardiovascular diseases. In a human study of carotid plaques, the intra plaque levels of omega-6 fatty acids and omega-3 fatty acids were evaluated in order to determine whether or not there is a decrease of these fatty acids in systematic patients with carotid plaques. Concentrations of these two fatty acids were evaluated using ELISA and it was found that there was a significant intra-plaque omega-3 fatty acids decrease in symptomatic patients. Omega-6 fatty acids levels did not show any differences relative to asymptomatic patients [33]. In the same year, another group showed that alpha-linolenic acid (ALA) was neuroprotective against ischemic brain damage. ALA is part of the essential fatty acids meaning that the human body cannot produce it and it needs to be acquired through diets. It was shown that after subchronic treatments of ALA, proteins such as synaptophysin-1, VAMP-2 and SNAP-25 were increased relative to the control diet group. It is very well documented that these proteins are essential for the formation and maintenance of synaptic activity. Also, there was a significant increase of V-GLUT1 and V-GLUT2 which are responsible for glutamate transmission and they may assist to protect the brain from glutamate excitotoxicity. Brain derived neurotrophic factor or BDNF was also increased, showing a positive correlation with anti-depressant activity in the experimental mice relative to the control diet mice. Moreover, ALA beneficial neuroprotective effects were tested in an ischemic stroke model. It was found that pre-treatment with ALA could reduce the post-ischemic infarct volume after 24 h of one hour of middle cerebral artery occlusion. Also, it was found that post-treatment of ALA after ischemic stroke insult could increase survival rates 10 days after the ischemic insult [34]. A very common dietary supplement used in China for the treatment of stroke is Ginseng. A group led by Zheng has been studying ginseng’s ability to increase neurogenesis and its effects in neurological effects. In a focal cerebral ischemia model, Ginseng total saponins (GTS) neuroprotection was examined. After three days of intraperitonial (IP) injection of either GTS or saline, male Wistar rats underwent permanent middle cerebral artery occlusion (MCAO). Animals were euthanized and BrdU, a thymidine analog and cell proliferation marker, NeuN, a mature neuronal marker and neurological scores were assessed. Results showed that GTS-treated rats performed better on neurological scores relative to control. The number of proliferation cells and the ratio of proliferating cells and mature neurons in the SVZ and in the ipsilateral to the infarct area were significantly higher than the control group. In sum, this study showed how GTS could not only induce the proliferation of neural stem cell but also their differentiation into functional neurons as shown by the increase of neurological scores in these rats [35]. Another dietary supplementation that has been used and prescribed in many countries due to it medicinal benefits in the brain, is the Ginkgo biloba. In a study led by Dores, a standardized Ginkgo extract called EGb761 was evaluated in a transient ischemia model in heme oxigenase 1 (HO-1) knockout mice. Heme oxigenase is an enzyme that has been associated with cytoprotection after oxidative stress [36]. Previously, it was shown by Dore’s group, that Ginkgo biloba is able to increase heme oxigenase. In this study, mice were pretreated acutely for seven days before MCAO and during reperfusion. It was found that the neuroprotective effects of the EGb761 were abolished on the HO-1 knockout mice. Supporting this notion, a neurobehavioral test showed HO-1 knockout mice neurologically dysfunctional compared with wild type mice. In sum, this study is an attempt to investigate the mechanism of action of Ginkgo biloba after an ischemic and oxidative stress insult, showing that Ginkgo biloba might exert its neuroprotection by increasing HO-1 which is cytoprotective against oxidative insults and stress [37].

The reverse diet manipulation paradigm involving caloric restriction and intermittent fasting has also been found to protect against stroke [29,30,38]. Here, rodents maintained on a caloric restriction diet or intermittently fasted, developed an increased resistance of brain cells to experimental models of stroke. Postulated mechanisms underlying the neuroprotection produced by restricted diet intake include decreased oxidative stress and increased cellular stress resistance which singly or in combination can promote a “preconditioning” state, priming the brain to withstand a stroke insult. Additionally, upregulation of neurotrophic factor (*i.e.*, increased BDNF levels) signaling pathways was observed [30]. Furthermore, these authors noted that diet restriction can stimulate neurogenesis and enhance synaptic plasticity [38,39]. These studies by [39] indicate that a regimen of reduced food intake exerts neuroprotection against stroke.

These two lines of laboratory evidence support our general hypothesis that diet manipulation, via supplementation of nutritious diet extracts/compounds or by restricting unhealthy diet (*i.e.*, high caloric intake), stands as beneficial stroke adjunct therapy. However, upon careful examination of these studies, it is clear that the mechanisms mediating the diet-induced neuroprotection warrant further examination. In our diet supplementation paradigm, we will pursue the neurogenesis mechanism, which is suggested by [39], and also in part because of our long-standing interest in stem cell therapy research [40,41].

### 1.4. Significance and Product Development

Our data and others, strongly support the diet supplementation paradigm model, to enhance neurogenesis in the adult rat stroke model due to its ability to provide nutrients that balance the homeostasis of the brain’s milieu. Moreover, our rationale for focusing on diet manipulation in elucidating the effects of nutritious diet on neurogenesis is largely based on the existing reverse paradigm of “unhealthy diet restriction” [39] as a neuroprotective strategy. The strategic scientific advance in this proposed study is our desire to provide a closer approximation of the diseased brain state (*i.e.*, after stroke), which is likely characterized by an imbalance of necessary nutrients in the brain microenvironment. Supporting evidence shows that exposure to unhealthy diets such as high fat diets are detrimental to the endogenous regenerative capacity after an ischemic insult. Also, after a long-term exposure to high fat diets, there is an increase of the pro-inflammatory cytokine TNF-α in the brain, activated migroglia, APP levels, and macrophage activation [42,43].

The diet supplementation paradigm will generate new information about neurogenesis in the stroke brain that should complement the diet restriction model, and advance the entire field of diet manipulation for stroke. We hypothesize here that our proposed diet supplementation strategy will achieve the desirable neuroprotection that increases cell proliferation beneficial to stroke outcome. This review paper makes a case in extending our scientific platform for determining the benefit of NT-020 diet supplementation in stroke. We will determine the optimal dosage and therapeutic window post-injury, and examine the mechanism underlying NT-020 neuroprotection in defining a clinical development strategy for adjunct treatment in ischemic stroke. This paper will establish important Phase I background information that will guide us to develop a Phase II study for further product development, safety studies and clinical trials.

## 2. Preclinical Studies

This section establishes the feasibility of achieving the goals of the proposed paradigm advancing the therapeutic potential of NT-020 supplementation as a post-stroke treatment based on the accomplishment of major discovery-driven milestones, namely, *in vivo* studies showing the therapeutic efficacy of NT-020 to induce neurogenesis and to reduce stroke-induced behavioral and histological deficits in a pre-injury treatment paradigm. These data, combined with the published reports of neuroprotective effects of individual dietary components of NT-020 in animal models of stroke [31], Parkinson’s disease [44], and aging [23], form the basis for proceeding with our desire to examine the therapeutic potential NT-020 in a post-stroke paradigm.

### 2.1. NT-020 Attenuates Stroke-Induced Behavioral Deficits

Two groups of adult male Sprague-Dawley rats initially received 135 mg/kg of NT-020 (*n* = 8) or vehicle (*n* = 7). The highest dosages of the components on NT-020 (135 mg/kg) were used and adjusted accordingly, for *in vivo* administration [40–42,45,46]. This high dose was previously evaluated and it was found to promote the greatest amount of cell proliferation in the *in vitro* cell studies [19]. Dosing for NT-020 and vehicle consisted of daily oral administration (using oral gavage) in addition to traditional rat chow over a two-week period. As indicated by [31], such pre-stroke diet supplementation dosing regimen should be within the therapeutic range to promote neuroprotection. On day 14 following the last treatment, all animals underwent the stroke surgery using the transient one-hour suture occlusion of middle cerebral artery (MCAo) [40,41]. To reveal the functional effects of NT-020, animals were subjected to established behavioral tests just prior to stroke surgery and again on day 14 post-stroke. Behavioral tests included Bederson test and elevated body swing test (EBST), which are both sensitive functional measures of stroke-induced motor and neurological deficits respectively.

**Figure 1:**
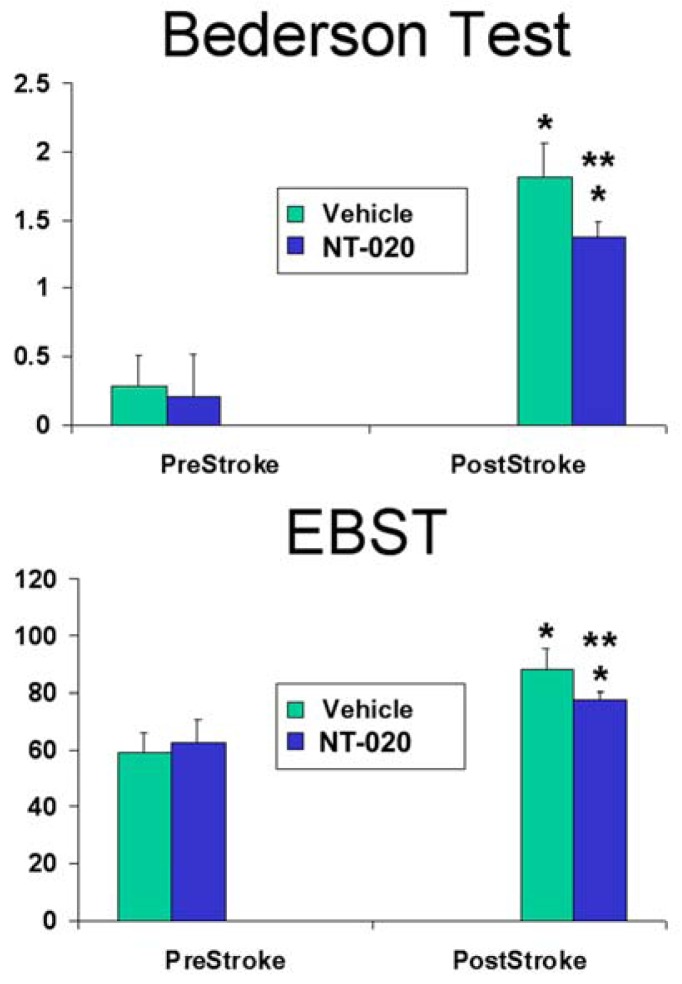
Neurologic (Bederson test) and motor (EBST) evaluations prior to stroke surgery (pre-stroke) revealed no detectable behavioral deficits between groups. At 14 days after stroke (post-stroke), while both groups exhibited motor and neurologic deficits (*versus* pre-stroke: * *p* < 0.05), the NT-020-treated rats exhibited significantly less motor and neurologic deficits than vehicle-treated rats (** *p* < 0.05). Reproduced from Rejuvenation Research [21].

ANOVA revealed significant treatment effects in both Bederson (*F*_3,26_ = 81.65, *p* < 0.0001) and EBST (*F*_3,26_ = 29.26, *p* < 0.0001) (Figure 1). Pair-wise comparisons between treatment groups using Fisher’s PLSD posthoc *t*-tests revealed NT-020- and vehicle-treated groups displayed no detectable behavioral impairments at pre-stroke testing, but both exhibited motor and neurologic deficits at post-stroke testing (compared to pre-stroke: *p* < 0.05). However, NT-020-treated stroke animals showed significantly less abnormalities in both motor and neurologic tests compared to vehicle-treated stroke animals (at post-stroke testing: *p* < 0.05). Reductions of 11.8% and 24.4% in EBST motor asymmetry and Bederson neurologic dysfunction, respectively, were detected in NT-020-treated stroke animals compared to vehicle-treated stroke animals. These results indicate that pre-stroke treatment with NT-020 significantly ameliorated motor and neurological abnormalities.

### 2.2. NT-020 Reduces Stroke-Induced Cerebral Infarction

**Figure 2:**
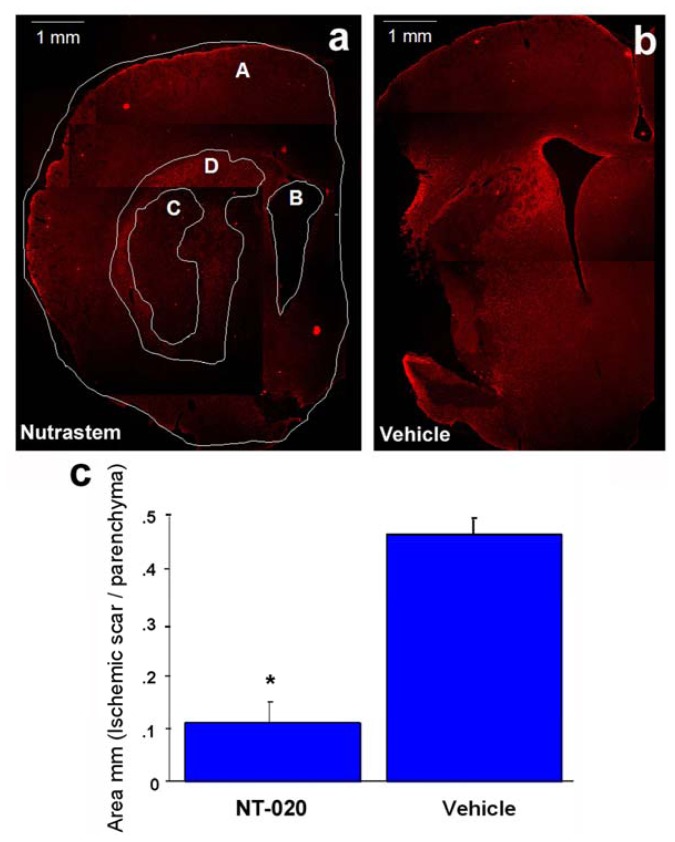
Glial fibrillary acidic protein (GFAP) immunostaining (see Methods for detailed protocol) was used to reveal glial scar. Panel a shows calculation of glial scar area using the formula C = D/(A − B), with A: total hemisphere area; B: lateral ventricle; C: glial scar; and D: penumbra. NT-020 treatment (**a**) significantly reduced the glial scar compared to vehicle treatment (**b**), as quantified in (**c**) (* *p* < 0.0005). Reproduced from Rejuvenation Research [21].

Following the behavioral testing at day 14 post-stroke, all animals were euthanized in order to evaluate the cerebral infarction using the glial fibrillary acidic protein (GFAP) immunostaining [47]. This routine GFAP assay reveals the extent of glial scar, which closely approximates the cerebral damage in stroke. Although triphenyltetrazolium chloride (TTC) is more routinely used to reveal cerebral infarction, such TTC assay is only sensitive when performed within seven days post-stroke. Using an NIH imaging system, the glial scar was calculated by capturing images using AxioPhot (Carl Zeiss) at 1.6-fold magnification. The damaged area was selected according to the morphology of the cells based on glial infiltration which clearly delineated the ischemic core from the ischemic penumbra [47]. The mean area of damage of 5–6 sections per coronal slice was calculated using the following formula: C = D/(A − B), to reveal the total infarct area per brain (see Figure 2).

Student *t*-test revealed that NT-020 reduced the glial scar/ischemic area damage in the striatum (Figure 2) compared to vehicle treatment (Figure 2). A significant 75% decrement in mean glial scar area was observed in the ischemic striatum of NT-020-treated stroke animals compared to that of vehicle-treated stroke animals (*p* < 0.0005) (Figure 2). These histological results parallel the behavioral performance of NT-020-treated stroke animals, suggesting that a reduction in the extent of cerebral infarction translated to attenuation of stroke-induced functional deficits.

### 2.3. NT-020 Induces Cell Proliferation in Stroke Brain

This next set of data was generated to determine whether the NT-020-induced cell proliferation seen *in vitro* also occurs *in vivo*, which could serve as the mechanistic explanation for the observed behavioral and histological protection. Alternate brain sections obtained from the same NT-020- or vehicle-treated stroke animals above were processed for BrdU immunostaining (Sigma, 50 mg/kg, i.p. every eight hours during days 10 to 14 post-stroke) to reveal cell proliferation. Analyses of BrdU labeling were focused at the neurogenic (SVZ) and the nonneurogenic striatum. The rationale for initially focusing on SVZ is to reveal that NT-020 could indeed increase cell proliferation in a neurogenic site. Although an increase in cell proliferation in SVZ could be used as a reasonable index to support a mechanism for NT-020-mediated neuroprotection, we further tested this hypothesis by subsequently analyzing BrdU labeling in the ischemic striatum to reveal whether newly formed cells from the SVZ are able to migrate towards the site of injury. This migration potential would reveal to us stronger evidence of NT-020’s therapeutic efficacy as such data would demonstrate the compound’s potential to not only increase cell proliferation, but also to facilitate proper homing of newly formed cells to the appropriate injured brain site.

**Figure 3:**
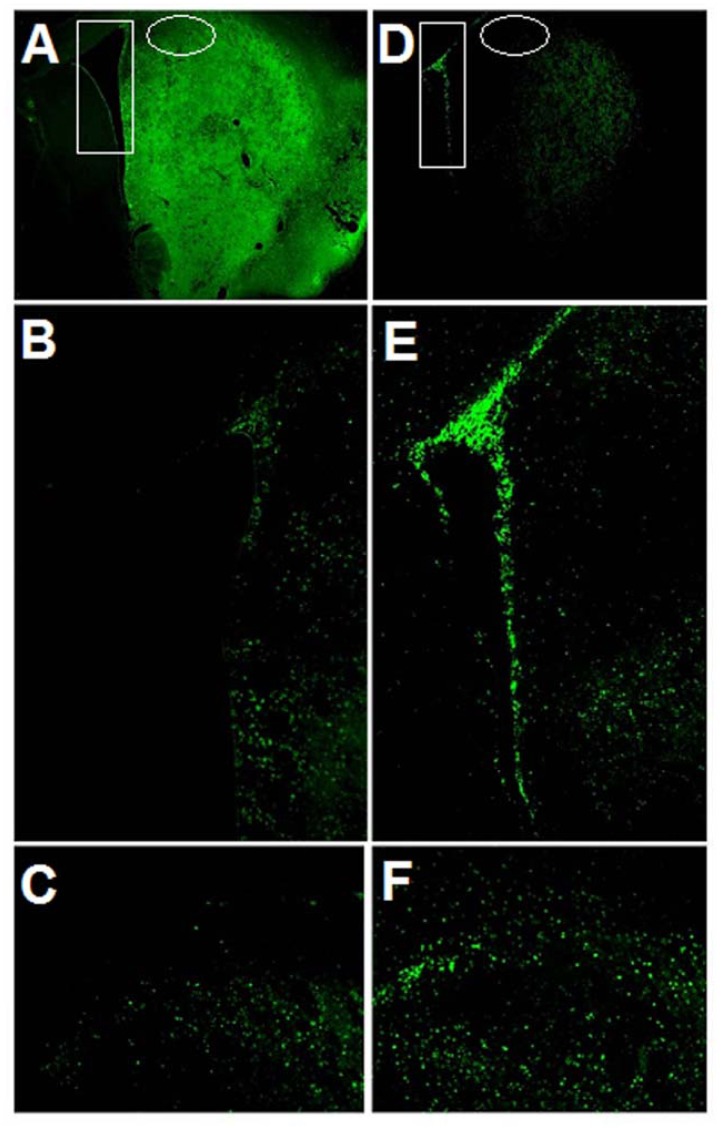
BrdU labeling (see Methods for detailed protocol) of brains from vehicle-treated (**A**–**C**) and NT-020-treated (**D**–**F**) stroke animals. NT-020 increased the number of BrdU-positive proliferating cells in neurogenic (SVZ) (**D** in rectangle and magnified in **E**) and ischemic striatum (**D** in circle and magnified in **F**) compared to vehicle treatment (SVZ: **A** in rectangle and magnified in **B**; striatum: **A** in circle and magnified in **C**). Reproduced from Rejuvenation Research [21].

**Figure 4:**
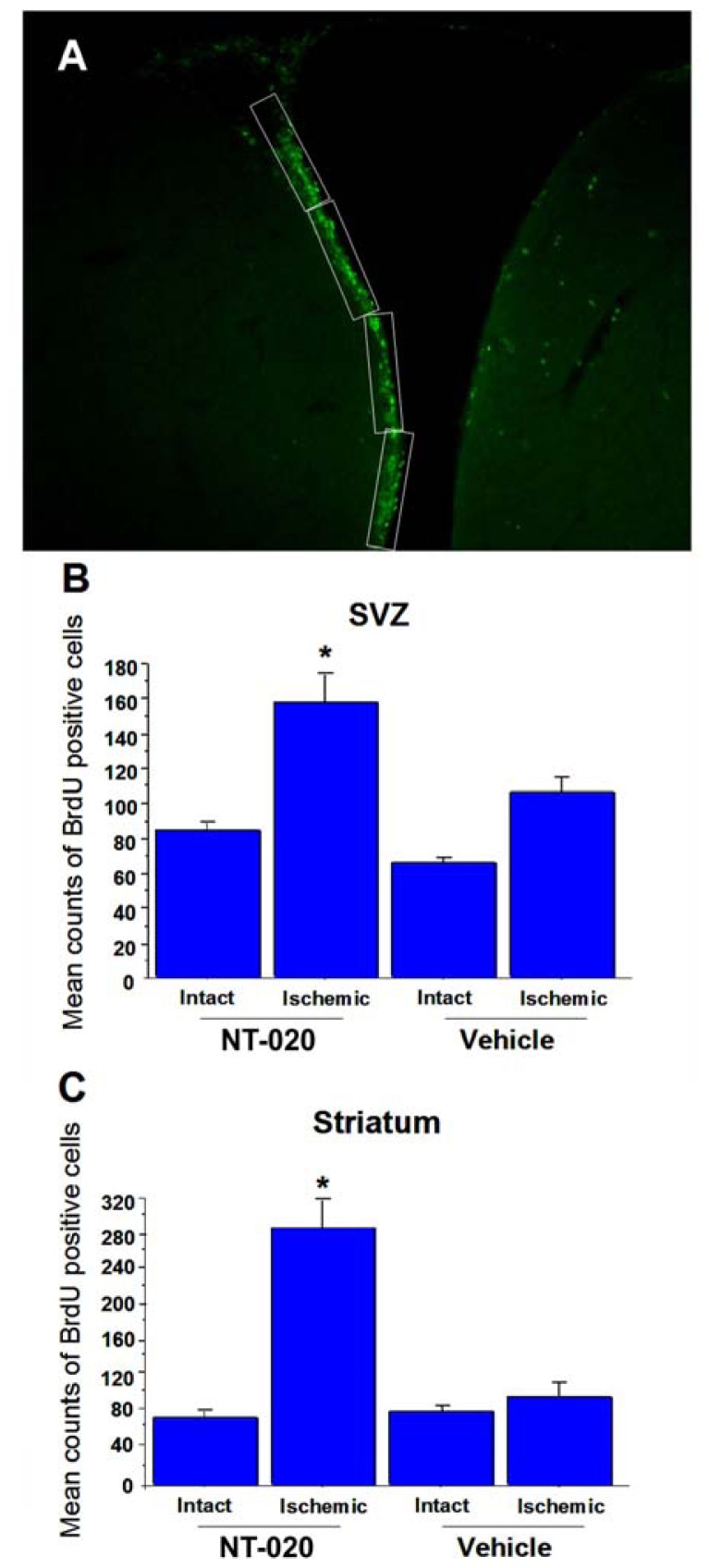
Quantitative analysis of BrdU labeling in SVZ (**A**) followed the protocol by [6]. Four rectangular sections (200 μm × 60 μm) at 100× magnification from two serial brain sections from each rat were used to reveal mean BrdU cell counts along the SVZ. For quantitative analysis of BrdU labeling in striatum, two serial brain sections from each rat capturing the striatal penumbra depicted in Figure 3A,D (magnified in Figure 3C,E), with each section corresponding to 40 μm × 40 μm, were used to reveal mean BrdU cell counts in the ischemic striatum. Results revealed one-fold and three-fold increments, respectively, in the SVZ (**B**) and striatum (**C**) of the NT-020-treated stroke brains compared to vehicle-treated stroke brains (* *p* < 0.0005 in SVZ and * *p* < 0.0001 in striatum when comparing corresponding ischemic SVZs and striata between treatment groups). Reproduced from Rejuvenation Research [21].

Immunofluorescence microscopy revealed NT-020 enhanced cell proliferation in SVZ and ischemic striatum characterized by significantly increased BrdU labeling in these brain sites compared to vehicle-treated stroke brains (Figure 3). Quantitative analysis of SVZ’s cell proliferative activity (Figure 4; protocol based on [6]) revealed significant treatment effects (*F*_3,16_ = 18.03, *p* < 0.0001), with at least a one-fold increment in the number of BrdU-positive cells in the NT-020-treated stroke brains compared to vehicle-treated stroke brains (*p* < 0.0005) (Figure 3A,D in rectangles and magnified in Figure 3B,E; quantitative data shown in Figure 4B). Similarly, quantitative analysis of BrdU labeling in the ischemic striatal penumbra revealed significant treatment effects (*F*_3,16_ = 11.84, *p* < 0.0001), with at least a three-fold increase in the number of BrdU-positive cells in the NT-020-treated stroke brains compared to vehicle-treated stroke brains (*p* < 0.0001) (Figure 3A,D in circles and magnified in Figure 3C,F; quantitative data shown in Figure 4C). In contrast, when evaluation of BrdU labeling targeted the ischemic core (instead of the penumbra, see Figure 3A,D), there was a massive proliferation of BrdU-positive cells in the vehicle-treated stroke brains compared to NT-020-treated stroke brains. This obvious increment in BrdU labeling in the vehicle-treated ischemic core is not surprising as it has been established that BrdU labels infiltrating reactive microglia, as well as degenerating or dead cells which are abundant in the necrotic core [47–50]. Since NT-020-treated stroke brains have smaller ischemic core, it is expected that BrdU labeling in this region is less than that of the vehicle-treated stroke brains. Accordingly, examination of cell proliferation in the ischemic core presents as an artifact and may not truly reflect the neuroprotective BrdU labeling index. With this in mind, we limited the evaluation of cell proliferation to SVZ and ischemic penumbra. Both these sets of data indicate that NT-020 increases cell proliferation in the neurogenic SVZ, and also facilitated the migration of several of these newly formed cells towards the ischemic striatal penumbra.

### 2.4. NT-020 Promotes Neurogenesis in Stroke Brain

**Figure 5:**
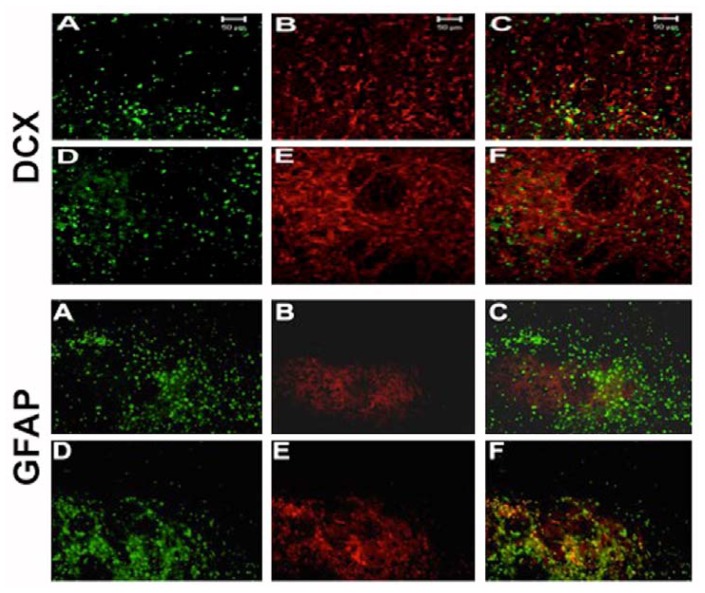
NT-020 enhanced neuronal, but not glial, differentiation. DCX: Newly formed cells in the ischemic striatum revealed by a high number of BrdU (**D**) and doublecortin (**E**) double-labeled cells (**F**) compared to vehicle treatment (**A**: BrdU; **B**: doublecortin; **C**: merged). GFAP: In contrast, NT-020 treated ischemic striatum showed very few cells positive for BrdU (**D**) and GFAP (**E**) that double-labeled (**F**). Of note, there were several cells that double-labeled with BrdU and GFAP in the vehicle treated ischemic striatum (**A**: BrdU; **B**: GFAP; **C**: merged). Reproduced from Rejuvenation Research [21].

**Figure 6:**
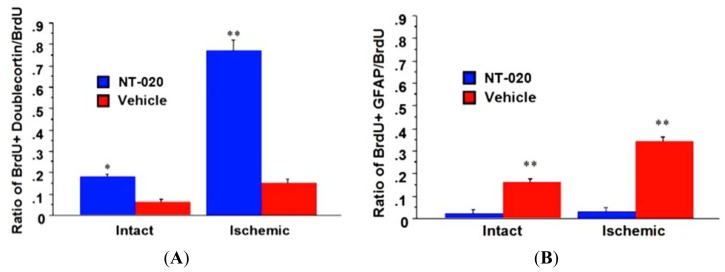
Quantitative analysis revealed significantly higher double-labeling of BrdU and doublecortin in NT-020 than vehicle-treated stroke brains (* *p* < 0.05, ** *p* < 0.0001) (**A**). In contrast, there were significantly lower BrdU and GFAP double-labeling in NT-020 than vehicle-treated stroke brains (** *p* < 0.0001) (**B**). Reproduced from Rejuvenation Research [21]. (**A**) (**B**)

In the same vein of determining the functionality of cell proliferation and migration, we further pursued characterizing whether NT-020’s enhancement of newly formed cells similarly lead to increased expression of neuronal phenotypes. Such neural differentiation, to us, is a much more stringent marker, than merely enhanced cell proliferation and migration, that would bolster our hypothesized mechanism of neurogenesis underlying NT-020 neuroprotection. BrdU-labeled brain sections (*i.e.*, ischemic striatal penumbra) used for cell migration studies above were double-labeled with the neuronal marker doublecortin or glial marker GFAP. Immunofluorescence microscopy revealed widespread double-labeling of cells with BrdU and doublecortin in NT-020-treated stroke brains, whereas only a few cells double-labeled with both BrdU and doublecortin in vehicle-treated stroke brains (Figure 5). Quantitative analysis revealed about 17% and 75% double-labeling of BrdU and doublecortin in respective intact and infarcted side of NT-020-treated stroke brains, which were significantly higher than those seen in the intact (5%) and infarcted side (13%) of vehicle-treated stroke brains (* *p* < 0.05, ** *p* < 0.0001) (Figure 6A). In contrast, only a few cells in NT-020-treated stroke brains double-labeled with BrdU and GFAP, while many cells in vehicle-treated stroke brains double-labeled with BrdU and GFAP. Quantitative analysis revealed about 1% and 2% double-labeling of BrdU and GFAP in respective intact and infarcted side of NT-020-treated stroke brains, which were significantly lower than those seen in the intact (18%) and infarcted side (35%) of vehicle-treated stroke brains (** *p* < 0.0001) (Figure 6B). These data indicate that NT-020 induces neural differentiation, with increased tendency towards neuronal over glial lineage. In view of the pathological manifestation of stroke, at least in this MCAo model, characterized by extensive neuronal loss accompanied by increased glial cell activation, it appears that neuronal replacement will be more beneficial than glial cell replenishment. The robust neuronal differentiation at two weeks post-stroke is equally advantageous since a rapid cell death cascade proceeds after the stroke onset. Thus, the preferential neuronal differentiation during the acute stroke phase provides a solid evidence that neurogenesis plays a major active role in the NT-020-mediated neuroprotection.

In summary, these data [21] indicate the remarkable neuroprotective effects of NT-020 when given prior to stroke, possibly via its neurogenic potential. We hypothesize, based upon the data observed, that NT-020 not only increases the basal neural stem cell proliferation in the niche, in this case the SVZ, but also recruits endogenous neural stem cells from the niche to the injured tissue, thus promoting aspects of neural repair. This host tissue repair also continues for some time after the administration of NT-020 as the diet was given prior to the insult, but not continued following the insult, indicating that perhaps NT-020 works via epigenetic modulation of neural stem cells and other cells, to increase neuroprotection. One other observation is that the synergy which had been noted in the development of NT-020 in cell culture experiments [19] is also observed *in vivo*. The dose of blueberry alone to produce neuroprotection in a previous study [31] is 100 times higher than used in this study. Based upon these encouraging data, we now advance a translational study of NT-020 in a post-stroke paradigm to further evaluate its efficacy in a clinically relevant animal model.

## 3. Caveats to Consider for Translational Research of NT-020

The overall design of our envisioned translational experiments is geared towards the examination of the neuroprotective effects of diet supplementation in a post-stroke paradigm. Three functional outcomes that are deemed critical to the demonstration of the optimal regimen of NT-020 for stroke include: (1) assessing behavioral neuroprotection of NT-020 by examining stroke-induced motor and neurologic functions of stroke animals; (2) evaluating histological neuroprotection of NT-020 by measuring stroke-induced cerebral infarction; and (3) identifying the mechanism underlying NT-020 neuroprotection by immunohistochemically characterizing newly formed cells or cells that are undergoing neurogenesis, as well as those cells differentiating into neurons, astrocytes or oligodendrocytes.

Guidance on the experimental design of this translational study draws largely from the demonstration that pre-stroke treatment of NT-020 attenuated stroke-induced behavioral and histological deficits [21]. The overarching hypothesis is that NT-020 post-stroke treatment will similarly produce significant functional effects relative to vehicle-treated stroke animals. The limiting factor is the demonstration of the appropriate NT-020 dosage post-stroke. Accordingly, translational studies should begin to examine NT-020 at a dosage found as optimal in the pre-stroke paradigm. However, for this post-stroke paradigm, there is anticipation of the rapid cell death cascade inherent with stroke pathology that will likely require high NT-020 dosage in the absence of the pre-treatment regimen. All the higher dosage formulations of NT-020 are still within clinically tolerable dosages based on other compounds in the market [51] and our previous studies [21,23]. Thus, these higher dosages of NT-020 will likely exert the most beneficial behavioral and histological effects. Although adverse side effects of NT-020 in all dosages are not expected, closely monitoring any unexpected harmful effects should be performed and may be manifested in the behavioral and histological assays.

The timing of the diet supplement spirulina may be effective up to 24 h post-stroke [21]. Capturing this therapeutic window post-stroke will provide guidance on the optimal treatment initiation regimen. A longer post-stroke time point may also allow identification of a treatment maximal limit. In this case, the hypothesis is that NT-020 when administered immediately and at some proximal period following MCAo stroke will produce optimal behavioral and histological benefits. The yardstick for determining equivalence of functional benefits across NT-020 treatment onset will be for stroke animals to display behavioral and histological neuroprotection comparable to those treated with NT-020 at the earliest time point (*i.e.*, 5-min delay). Although it is possible that a short-delay (1–3 h) post-stroke may prove more beneficial than the 5-min delay, the functional effects of NT-020 may be time-dependent, in that administration of the compound at long delay post-stroke will display waning of beneficial effects. However, based on our data [21], NT-020 remarkably enhances neurogenesis, which would suggest that even when NT-020 is administered after a long delay post-stroke, it is possible to observe functional benefits once “neurogenesis” becomes functional, *i.e.*, newly formed cells have migrated to the site of injury and have differentiated into neuronal cells. Thus, NT-020 treatments proximal to stroke onset will likely produce robust behavioral effects compared to long delay NT-020 treatments, but if neurogenesis indeed plays a significant role in NT-020 neuroprotection, the delayed NT-020 treatments will also produce functional effects at later post-stroke period of behavioral evaluation.

For assessment of mechanism of action, there is a need to recognize that stroke compromises BBB [40,41,52,53] and that aberrant immune response and detrimental inflammation ensue after the ischemic injury [54,55], further exacerbating stroke-induced neuropathological deficits. Our group has been particularly interested in the repair of BBB following injury (e.g., ALS and stroke) as a therapeutic strategy in retarding or even halting the disease progression [20,56–58]. Accordingly, incorporating assays designed to evaluate the BBB status may reveal unique mechanisms of action for NT020. Indeed, recent publications from our team reveal that dietary supplementation exerts anti-inflammatory effects [59] which could be related to BBB reconstitution. This assessment of stroke-induced BBB breakdown [40,41], as well as inflammation [18,60] thereby provide additional insights on possible interaction of NT-020’s anti-inflammatory effects and BBB repair.

In addition to anti-inflammatory effects and BBB repair, NT-020 at optimal dosage and optimal treatment onset is thought to afford neurogenesis as a major pathway of its neuroprotection. In particular, NT-020 treatment is envisioned to significantly enhance neurogenesis, characterized by increased neurogenesis at least in the SVZ and in the ischemic striatum, compared to vehicle-treated stroke animals. Based on published data [21], pre-stroke NT-020 facilitates preferential neural differentiation with widespread expression of neuronal phenotypic marker over glial lineage. Along these lines, there is a high likelihood that post-stroke NT-020 will also promote neuronal over glial lineage differentiation.

While the long-term goal is to develop NT-020 as an adjunct agent, the short-term goal is to focus solely on evaluating the efficacy of NT-020 alone. Once NT-020 neuroprotection in the post-stroke paradigm is validated, subsequent experiments can proceed by assessing combination of NT-020 with the established stroke treatment of tPA. A clinical scenario wherein NT-020 extends the therapeutic window of tPA beyond three hours is desirable. In addition, combining NT-020 with experimental therapies, such as stem cell transplantation and neurotrophic factor therapy, may enhance the therapeutic benefits of these strategies for stroke.

The behavioral evaluation of NT-020 may initially focus on motor and neurologic tasks, as these simple tasks will be the most sensitive assays if NT-020 does indeed show neuroprotection. Following demonstration of amelioration of stroke-induced motor and neurologic impairments, subsequent evaluations may focus on NT-020’s potential to attenuate stroke-induced cognitive deficits, using tasks such as Morris water maze and radial arm maze. Indeed, aged rats fed with diet supplements display improvement in cognitive function [3]. In a study by [3], cumulative distance to platform was analyzed in the spatial navigation task Morris water maze. Within the aged impaired rats (AI), it was found that AI rats fed with NT-020 score lower distances when finding the platform in compared with AI fed with control diet. It was concluded that NT-020 supplementation ameliorated the spatial navigation impairment seen in aged impaired rats fed with control diet.

The clinical relevance of a post-stroke paradigm in testing the efficacy of NT-020 cannot be understated. Although the optimal diet supplementation regimen in the clinic is to treat prophylactically patients who are at risk of stroke and also to continue such treatment after stroke, evaluating NT-020’s efficacy in a more stringent stroke model, *i.e.*, post-stroke treatment should offer direct clinical applications. If NT-020 is proven effective when only delivered at post-stroke periods, this will extend NT-020 as a prophylactic agent to being an adjunct treatment in stroke patients who have not used diet supplementation prior to stroke. Since most stroke patients are likely not taking diet supplementation prior to stroke, testing the efficacy of NT-020 in such post-stroke paradigm will provide a solid evidence of its clinical utility as an adjunct treatment. In addition, in order to ensure the highest quality of NT-020 and batch-to-batch consistency, a GMP-licensed manufacturing facility has been contracted to produce quality-controlled and quality-assured clinical grade dietary supplement.

Equally as important as demonstrating efficacy, monitoring any potential adverse effects in the higher dosages of NT-020 will be clinically relevant. However, a wider range of dosages will be necessary to fully determine the safety and toxicity of NT-020. Such safety and toxicity studies can be addressed after determination of NT-020 efficacy in the post-stroke paradigm. To this end, we refer to our recent call, resonating the position of several others in the field, to adhere to STAIR (Stroke Therapy Academic Industry Roundtable) and STEPS (Stem cell Therapeutics as an Emerging Paradigm in Stoke) [34,61,62] criteria in therapeutics development for stroke, in particular the need to test experimental therapies in a clinically relevant animal model, such as the post-stroke paradigm and permanent MCAo and MCAl models, that should closely approximate the clinical safety and efficacy of any experimental treatment for stroke.

## 4. Conclusions

Finding a novel therapy for stroke is an urgent clinical need. This review article provides scientific evidence that dietary supplementation may afford neuroprotection for stroke. Whereas the laboratory evidence supports a pre-stroke treatment with dietary supplementation for producing neuroprotection in stroke, extending this treatment at post-stroke period will increase its clinical relevance. A systematic evaluation in a post-stroke model will help determine the optimal dosage and therapeutic window of NT-020 as a dietary supplement for stroke therapy. A further understanding on the mechanisms of action underlying dietary supplementaion, notably via anti-inflammatory and pro-neurogenic pathways will also facilitate in optimizing the treatment regimen. The use of dietary supplementation as an adjunct therapy is likely to improve stroke outcome.
